# Comprehensive analysis of DNA methylation and gene expression profiles in cholangiocarcinoma

**DOI:** 10.1186/s12935-019-1080-y

**Published:** 2019-12-26

**Authors:** Cheng Zhang, Bingye Zhang, Di Meng, Chunlin Ge

**Affiliations:** 1 Department of Pancreatic and Biliary Surgery, The First Hospital of China Medical University, Shenyang, 110001 Liaoning China; 2 Department of Gerontology, The First Hospital of China Medical University, Shenyang, 110001 Liaoning China

**Keywords:** Cholangiocarcinoma, Methylation, Bioinformatics

## Abstract

**Background:**

The incidence of cholangiocarcinoma (CCA) has risen in recent years, and it has become a significant health burden worldwide. However, the mechanisms underlying tumorigenesis and progression of this disease remain largely unknown. An increasing number of studies have demonstrated crucial biological functions of epigenetic modifications, especially DNA methylation, in CCA. The present study aimed to identify and analyze methylation-regulated differentially expressed genes (MeDEGs) involved in CCA tumorigenesis and progression by bioinformatics analysis.

**Methods:**

The gene expression profiling dataset (GSE119336) and gene methylation profiling dataset (GSE38860) were obtained from the Gene Expression Omnibus (GEO) database. Differentially expressed genes (DEGs) and differentially methylated genes (DMGs) were identified using the limma packages of R and GEO2R, respectively. The MeDEGs were obtained by overlapping the DEGs and DMGs. Functional enrichment analyses of these genes were then carried out. Protein–protein interaction (PPI) networks were constructed using STRING and visualized in Cytoscape to determine hub genes. Finally, the results were verified based on The Cancer Genome Atlas (TCGA) database.

**Results:**

We identified 98 hypermethylated, downregulated genes and 93 hypomethylated, upregulated genes after overlapping the DEGs and DMGs. These genes were mainly enriched in the biological processes of the cell cycle, nuclear division, xenobiotic metabolism, drug catabolism, and negative regulation of proteolysis. The top nine hub genes of the PPI network were F2, AHSG, RRM2, AURKB, CCNA2, TOP2A, BIRC5, PLK1, and ASPM. Moreover, the expression and methylation status of the hub genes were significantly altered in TCGA.

**Conclusions:**

Our study identified novel methylation-regulated differentially expressed genes (MeDEGs) and explored their related pathways and functions in CCA, which may provide novel insights into a further understanding of methylation-mediated regulatory mechanisms in CCA.

## Background

Cholangiocarcinoma (CCA) is a fatal malignancy originating from the epithelial cells of the biliary tree [1]. According to its anatomical location, the most contemporary classification of CCA includes intrahepatic, perihilar, and distal subtypes [2]. Surgical resection remains the only potentially curative treatment for all subtypes [1]. Unfortunately, early diagnosis is rare due to a lack of specific clinical symptoms [3]. Only about one-third of CCA patients are eligible for surgery; most patients are often diagnosed with unresectable or metastatic disease [4]. Even so, the recurrence and progression to distant metastasis are common within 2 years of resection [5]. Overall survival for patients with unresectable CCA does not exceed 14 months, even with systemic chemotherapy [6]. In addition, the incidence of CCA seems to be on the rise, especially for intrahepatic tumors [6]. Therefore, cumulative cholangiocarcinoma mortality rates have also increased by 39% [2]. Thus, exploring the molecular mechanisms underlying the pathogenesis and development of CCA is urgently needed.

DNA methylation is a central epigenetic modification that plays a key role in cellular processes, such as genome regulation, organism development, and disease [7]. Importantly, the dysregulation of DNA methylation patterns has been increasingly recognized as an important cellular event during both the initiation and late stages of oncogenesis [8, 9]. To date, numerous studies have demonstrated a crucial role for both hypermethylation of tumor suppressor genes and global hypomethylation of oncogenes in cancer development and progression, including in CCA [8, 10]. For instance, Chen et al. [11] found that the O6-methylguanine-DNA methyltransferase (MGMT) promoter was highly methylated, and the expression level of MGMT was positively correlated with overall survival rates and histological grade in CCA. In addition, the aberrant methylation of GATA-5, ANGPTL4, and DLEC1 has been shown to participate in the initiation and progression of this disease [12–14]. Despite the identification of several individual genes with specific hypomethylation or hypermethylation in CCA, comprehensive network studies based on methylation profiles and related pathways for these genes have been largely inadequate.

Over the past decade, gene profiling and next-generation sequencing technology have emerged as indispensable tools for cancer studies because they allow the detection of cancer-related genetic and epigenetic alterations, such as mutations, copy number variations, and DNA methylation changes across more extensive genomic regions [15, 16]. The bioinformatics analysis of these data can provide valuable information for CCA research. For example, Kong et al. identified three differentially expressed genes (DEGs), UCA1, miR-122, and CLIC1, based on next-generation sequencing data in CCA [17]. Analysis of these dysregulated genes showed that they could promote CCA progression through the regulation of miR-122/CLIC1 and activation of the ERK/MAPK signaling pathway [17]. Furthermore, both Farshidfar et al. and Jusakul et al. identified many differentially methylated genes (DMGs) by methylation profiling or whole-genome sequencing that contributed to cholangiocarcinogenesis and might serve as methylation biomarkers in CCA [18, 19]. However, separate DEG and DMG analysis from a single study are limited, and multiple overlapping available datasets may provide more accurate and reliable clues through comprehensive bioinformatics analysis [20]. Thus far, there is still a lack of conjoint analysis involving both gene expression and methylation profiling microarray datasets in CCA.

In the present study, we performed an integrated bioinformatics analysis based on gene expression profiling by high-throughput sequencing (GSE119336) and gene methylation profiling microarray (GSE38860). The methylation-regulated differentially expressed genes (MeDEGs) were identified and then subjected to enrichment analysis. Moreover, we constructed a protein–protein interaction (PPI) network to identify hub genes to screen novel biomarkers and therapeutic targets in CCA for future research.

## Methods

### RNA-Seq and microarray data

We obtained the gene expression profiling dataset generated by high-throughput sequencing (GSE119336) and the microarray-based gene methylation profiling dataset (GSE38860) from the publicly available Gene Expression Omnibus database (GEO, https://www.ncbi.nlm.nih.gov/geo/). The expression dataset (GSE119336) contained 15 pairs of CCA tumors and matched non-tumor tissues, which was based on the GPL11154 platform (Illumina HiSeq 2000). The DNA methylation dataset (GSE38860), which was generated on the GPL8490 platform (Illumina HumanMethylation27 BeadChip), included a total of 28 primary CCA tissues and six matched adjacent normal tissues.

### Identification of MeDEGs

GEO2R (http://www.ncbi.nlm.nih.gov/geo/geo2r/) is an online analysis tool that allows users to compare two or more groups of samples in a GEO Series to identify deregulated genes under specific experimental conditions. In this study, GEO2R was adopted to screen for differentially methylated genes (DMGs). |t| > 2 and P < 0.05 were considered statistically significant. In addition, differentially expressed genes (DEGs) were identified using the limma R package with a threshold of |log_2_FoldChange| > 2 and P < 0.05. Using the lookup function (VLOOKUP) of excel, we overlapped the GSE38860 and GSE119336 datasets. Finally, hypomethylation-high expression genes were obtained after superimposition of upregulated and hypomethylated genes, and hypermethylation-low expression genes were obtained after superimposition of downregulated and hypermethylated genes. The hypomethylation-high expression genes and hypermethylation-low expression genes were identified as methylation-regulated differentially expressed genes (MeDEGs).

### Functional enrichment analysis

Gene Ontology (GO) annotation and pathway analysis of the MeDEGs were conducted using The Search Tool for the Retrieval of Interacting Genes (STRING, https://string-db.org/) [21], Kyoto Encyclopedia of Genes and Genomes (KEGG, https://www.kegg.jp/), and Reactome (https://reactome.org/). Statistically significant thresholds were set at a P < 0.05.

### PPI network construction, module analysis, and identification of hub genes

The PPI network of hypomethylation-high expression genes and hypermethylation-low expression genes was constructed using the STRING online database. An interaction score of > 0.4 and P < 0.05 were regarded as statistically significant. Cytoscape (version 3.6.1; http://www.cytoscape.org/) software was utilized to visualize the network. Molecular Complex Detection (MCODE), an app in Cytoscape, was used to screen modules within the PPI network with a standard of combined scores > 3 and nodes ≥ 5. Hub genes were identified using the CytoHubba in Degree-ranked method with a selection criterion for hub genes set at a degree score of greater than 16.

### Validation of hub genes

MEXPRESS (http://mexpress.be) is a straightforward and easy-to-use web tool for the integration and visualization of expression and DNA methylation based on The Cancer Genome Atlas (TCGA) database at a single-gene level [22, 23]. We used MEXPRESS to validate hypomethylation-high expression genes and hypermethylation-low expression genes. The cBioPortal (http://cbioportal.org) is an open-access resource for interactive exploration of multidimensional cancer genomics datasets that provides access to data for more than 5000 tumor samples from 20 cancer studies [24]. The cBioPortal was used to explore the genetic alterations of hub genes.

## Results

### Identification of MeDEGs in CCA

**Fig. 1:**
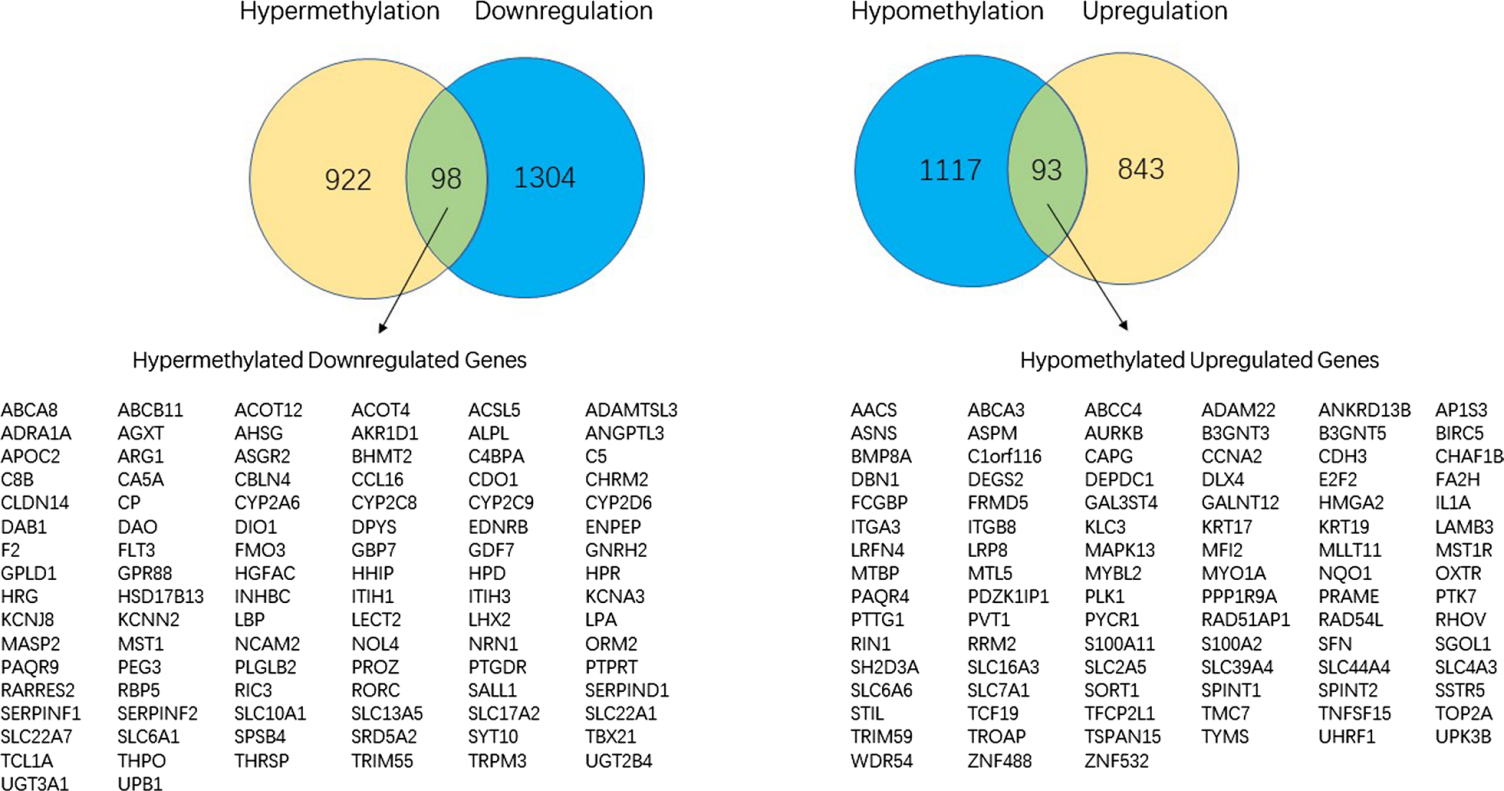
Identification of methylation-regulated differentially expressed genes (MeDEGs)

**Fig. 2:**
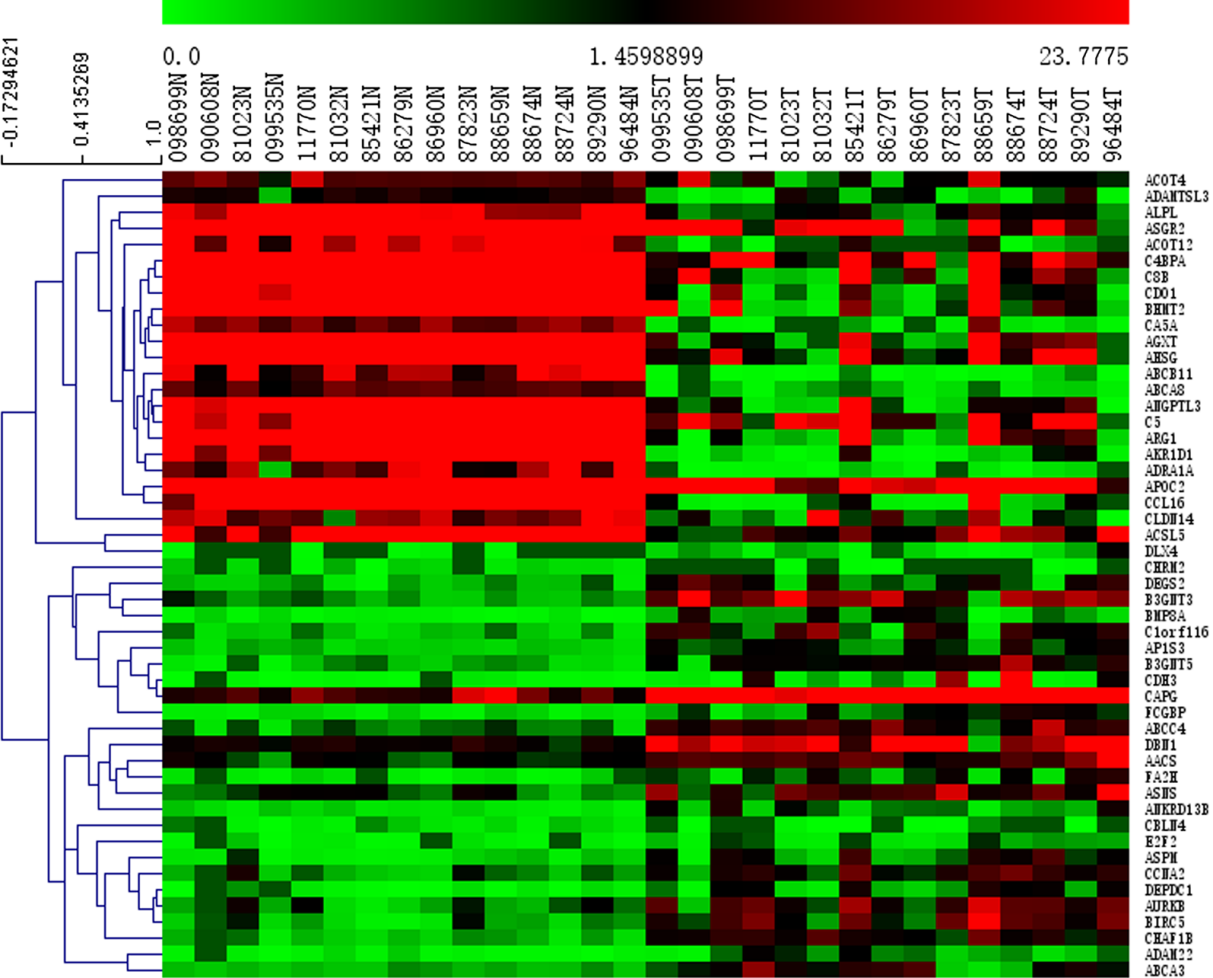
Heat map of the top 50 MeDEGs. X-axis represents samples, Y-axis represents genes, red stands for upregulation, and green stands for downregulation

The limma packages of R and GEO2R were used to screen for DEGs and DMGs, respectively. For the gene expression dataset GSE119336, a total of 2338 DEGs were identified, comprising 936 upregulated genes and 1402 downregulated genes. For the DMGs in the gene methylation dataset (GSE38860), 1020 hypermethylated genes and 1210 hypomethylated genes were obtained. As shown in Fig. 1, we identified 98 hypermethylated, downregulated genes and 93 hypomethylated, upregulated genes after the overlapping of the DEGs and DMGs. The heat map of the top 50 MeDEGs is shown in Fig. 2.

### Functional enrichment analysis of MeDEGs

The results of the gene ontology enrichment analysis for the MeDEGs are shown in Table 1. In the biological process group, hypomethylated, upregulated genes were mainly enriched in the cell cycle, nuclear division, negative regulation of cell cycle process, cell cycle process, and negative regulation of mitotic nuclear division. The hypermethylated, downregulated genes were mainly enriched in xenobiotic metabolic process, drug catabolic process, negative regulation of proteolysis, acute-phase response, and monocarboxylic acid metabolic process. We also found that the hypermethylated, downregulated genes were related to endopeptidase inhibitor activity, enzyme inhibitor activity, and serine-type endopeptidase inhibitor activity in the molecular function group and extracellular region in the cellular component group. Pathway enrichment was also performed using KEGG and Reactome, and the results are presented in Table 2. We found that hypermethylated genes predominantly participated in metabolism-related pathways, including general metabolic pathways, drug or xenobiotic metabolism by cytochrome P450, and cholesterol metabolism. For hypomethylated genes, the most significantly enriched pathways involved the cell cycle (mitotic G1-G1/S phases; G0 and early G1), SLC-mediated transmembrane transport, and signaling by MST1.

**Table 1:** Gene ontology enrichment analysis of MeDEGs

Category	Term ID	Term description	Count	False discovery rate
Hypermethylated downregulated genes				
BP_FAT	GO:0019752	Carboxylic acid metabolic process	22	5.98E−07
BP_FAT	GO:0071466	Cellular response to xenobiotic stimulus	9	8.39E−05
BP_FAT	GO:0006805	Xenobiotic metabolic process	7	8.00E−04
BP_FAT	GO:0042737	Drug catabolic process	7	8.00E−04
BP_FAT	GO:0045861	Negative regulation of proteolysis	11	8.00E−04
BP_FAT	GO:0006953	Acute-phase response	5	1.30E−03
BP_FAT	GO:0010951	Negative regulation of endopeptidase activity	9	1.30E−03
BP_FAT	GO:0032787	Monocarboxylic acid metabolic process	12	1.30E−03
BP_FAT	GO:0001676	Long-chain fatty acid metabolic process	6	3.30E−03
BP_FAT	GO:0044281	Small molecule metabolic process	23	3.30E−03
CC_FAT	GO:0005576	Extracellular region	38	2.40E−08
CC_FAT	GO:0005615	Extracellular space	25	2.40E−08
CC_FAT	GO:0044421	Extracellular region part	28	2.40E−08
CC_FAT	GO:0044432	Endoplasmic reticulum part	19	1.30E−03
MF_FAT	GO:0004866	Endopeptidase inhibitor activity	9	1.20E−04
MF_FAT	GO:0004857	Enzyme inhibitor activity	11	4.20E−04
MF_FAT	GO:0004867	Serine-type endopeptidase inhibitor activity	6	7.80E−04
MF_FAT	GO:0008395	Steroid hydroxylase activity	4	1.90E−03
MF_FAT	GO:0015370	Solute:sodium symporter activity	5	3.60E−03
MF_FAT	GO:0016491	Oxidoreductase activity	13	3.60E−03
Hypomethylated upregulated genes				
BP_FAT	GO:0007049	Cell cycle	20	3.20E−03
BP_FAT	GO:0000280	Nuclear division	8	1.61E−02
BP_FAT	GO:0010948	Negative regulation of cell cycle process	8	1.61E−02
BP_FAT	GO:0022402	Cell cycle process	15	1.61E−02
BP_FAT	GO:0045839	Negative regulation of mitotic nuclear division	4	1.61E−02
BP_FAT	GO:0045930	Negative regulation of mitotic cell cycle	8	1.61E−02
BP_FAT	GO:0048519	Negative regulation of biological process	41	1.61E−02
BP_FAT	GO:0051726	Regulation of cell cycle	17	1.61E−02
BP_FAT	GO:1903047	Mitotic cell cycle process	12	1.61E−02
BP_FAT	GO:0007093	Mitotic cell cycle checkpoint	6	1.66E−02
BP_FAT	GO:0051716	Cellular response to stimulus	46	3.22E−02
BP_FAT	GO:0007568	Aging	7	3.23E−02
BP_FAT	GO:0031577	Spindle checkpoint	3	3.23E−02
BP_FAT	GO:0051302	Regulation of cell division	6	3.23E−02
BP_FAT	GO:2000816	Negative regulation of mitotic sister chromatid separation	3	3.23E−02
BP_FAT	GO:0007010	Cytoskeleton organization	13	3.36E−02
BP_FAT	GO:0007051	Spindle organization	5	3.36E−02
BP_FAT	GO:0007052	Mitotic spindle organization	4	3.36E−02
BP_FAT	GO:0007346	Regulation of mitotic cell cycle	10	3.36E−02
BP_FAT	GO:0009888	Tissue development	18	3.36E−02

**Table 2:** Pathway enrichment analysis of MeDEGs

Term ID	Term description	Count	False discovery rate
Hypermethylated downregulated genes			
KEGG:hsa04610	Complement and coagulation cascades	7	2.39E−05
KEGG:hsa00982	Drug metabolism—cytochrome P450	6	8.49E−05
KEGG:hsa04976	Bile secretion	6	8.49E−05
KEGG:hsa04979	Cholesterol metabolism	4	3.50E−03
KEGG:hsa00830	Retinol metabolism	4	7.10E−03
KEGG:hsa00980	Metabolism of xenobiotics by cytochrome P450	4	9.00E−03
KEGG:hsa01100	Metabolic pathways	16	9.00E−03
KEGG:hsa00983	Drug metabolism—other enzymes	4	9.30E−03
KEGG:hsa05020	Prion diseases	3	9.30E−03
KEGG:hsa05204	Chemical carcinogenesis	4	9.30E−03
Reactome:HSA-1430728	Metabolism	28	1.10E−04
Reactome:HSA-211999	CYP2E1 reactions	4	1.10E−04
Reactome:HSA-9027307	Biosynthesis of maresin-like SPMs	3	6.50E−04
Reactome:HSA-114608	Platelet degranulation	6	1.70E−03
Reactome:HSA-76002	Platelet activation, signaling and aggregation	8	1.70E−03
Reactome:HSA-425366	Transport of bile salts and organic acids, metal ions and amine compounds	5	2.00E−03
Reactome:HSA-211859	Biological oxidations	7	2.30E−03
Reactome:HSA-977606	Regulation of complement cascade	4	2.30E−03
Reactome:HSA-373076	Class A/1 (Rhodopsin-like receptors)	8	2.90E−03
Reactome:HSA-211945	Phase I—functionalization of compounds	5	3.10E−03
Hypomethylated upregulated genes			
KEGG:hsa04110	Cell cycle	5	4.16E−02
Reactome:HSA-8852405	Signaling by MST1	3	1.50E−03
Reactome:HSA-1640170	Cell cycle	11	1.08E−02
Reactome:HSA-453279	Mitotic G1-G1/S phases	6	1.08E−02
Reactome:HSA-69278	Cell cycle, mitotic	10	1.08E−02
Reactome:HSA-1538133	G0 and Early G1	3	2.07E−02
Reactome:HSA-174143	APC/C-mediated degradation of cell cycle proteins	4	2.54E−02
Reactome:HSA-425366	Transport of bile salts and organic acids, metal ions and amine compounds	4	2.54E−02
Reactome:HSA-6806834	Signaling by MET	4	2.54E−02
Reactome:HSA-6806942	MET receptor activation	2	2.54E−02
Reactome:HSA-425407	SLC-mediated transmembrane transport	6	2.74E−02

### PPI network construction, module analysis, and identification of hub genes

**Fig. 3:**
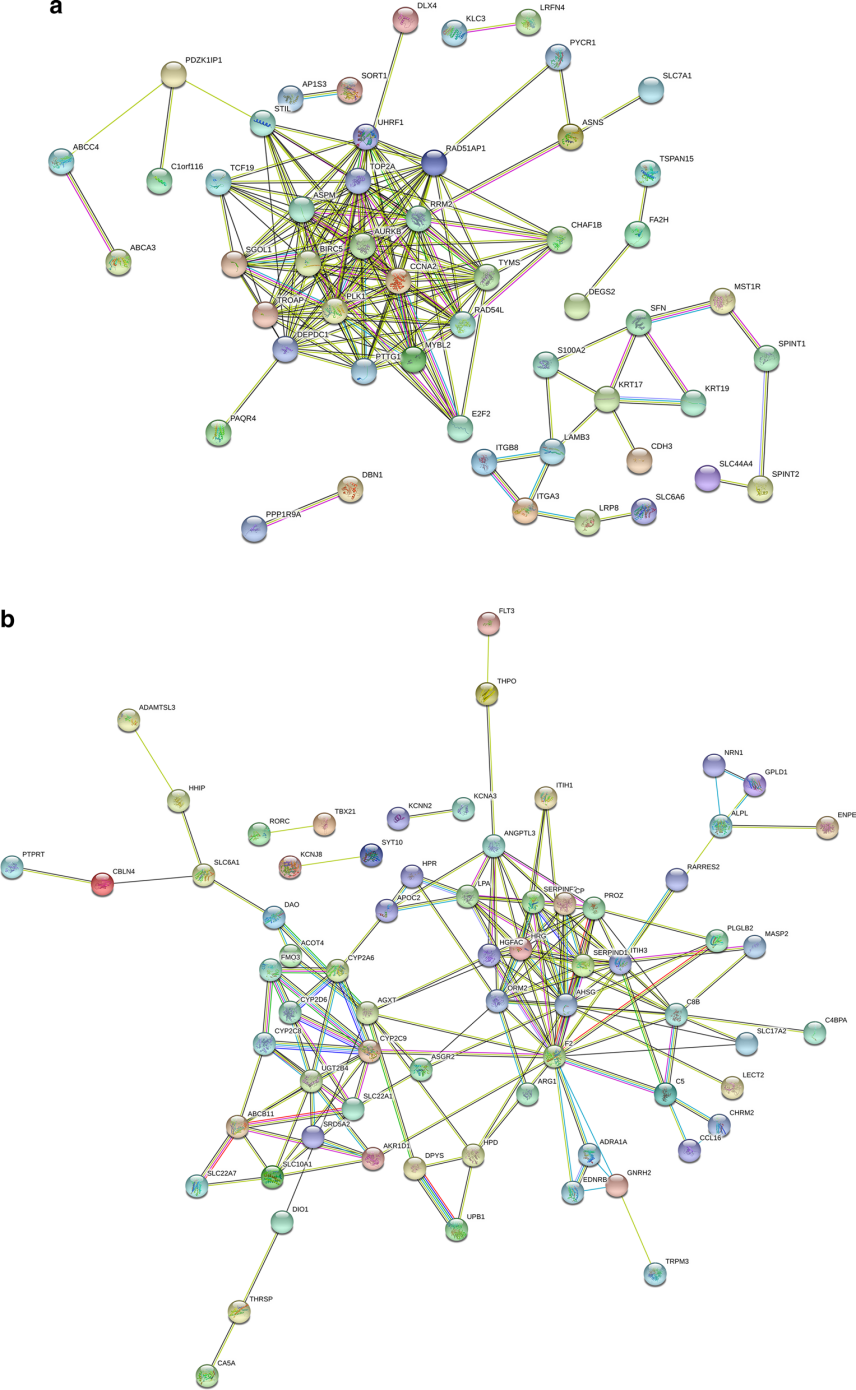
PPI network of methylation-regulated differentially expressed genes. **a** Hypomethylated upregulated genes. **b** Hypermethylated downregulated genes

**Fig. 4:**
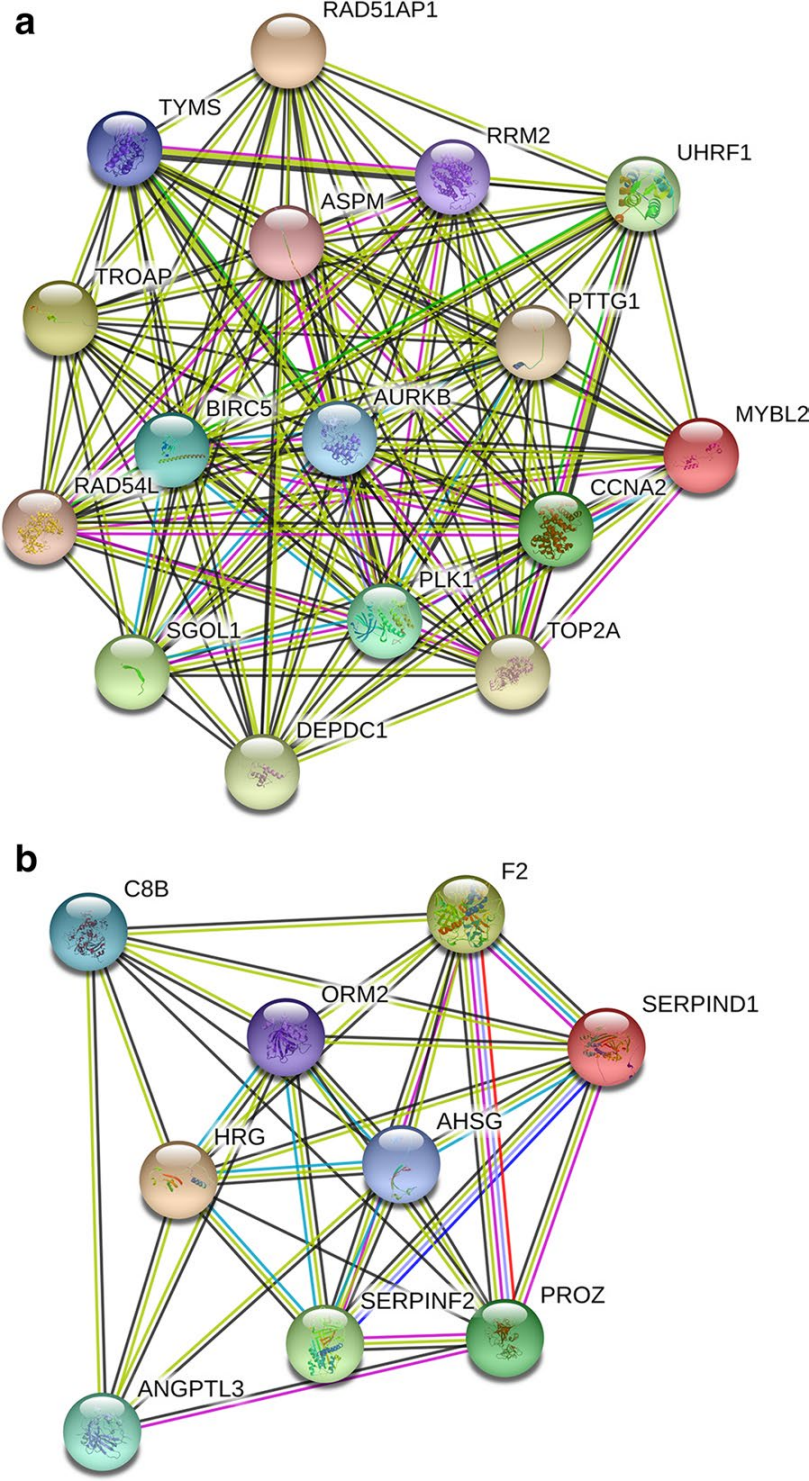
Module analysis of methylation-regulated differentially expressed genes. **a** Hypomethylated upregulated genes. **b** Hypermethylated downregulated genes

The PPI network of hypomethylation-high expression genes and hypermethylation-low expression genes was constructed using the STRING online database. An interaction score of > 0.4 and P < 0.05 indicated statistical significance. The results are presented in Fig. 3. Using MCODE in Cytoscape, we identified two modules in the PPI network with a standard of combined scores > 3 and nodes ≥ 5 (Fig. 4). Nine hub genes were identified using CytoHubba with a degree score greater than 16, including F2, AHSG, RRM2, AURKB, CCNA2, TOP2A, BIRC5, PLK1, and ASPM.

### Verification of hub genes

**Fig. 5:**
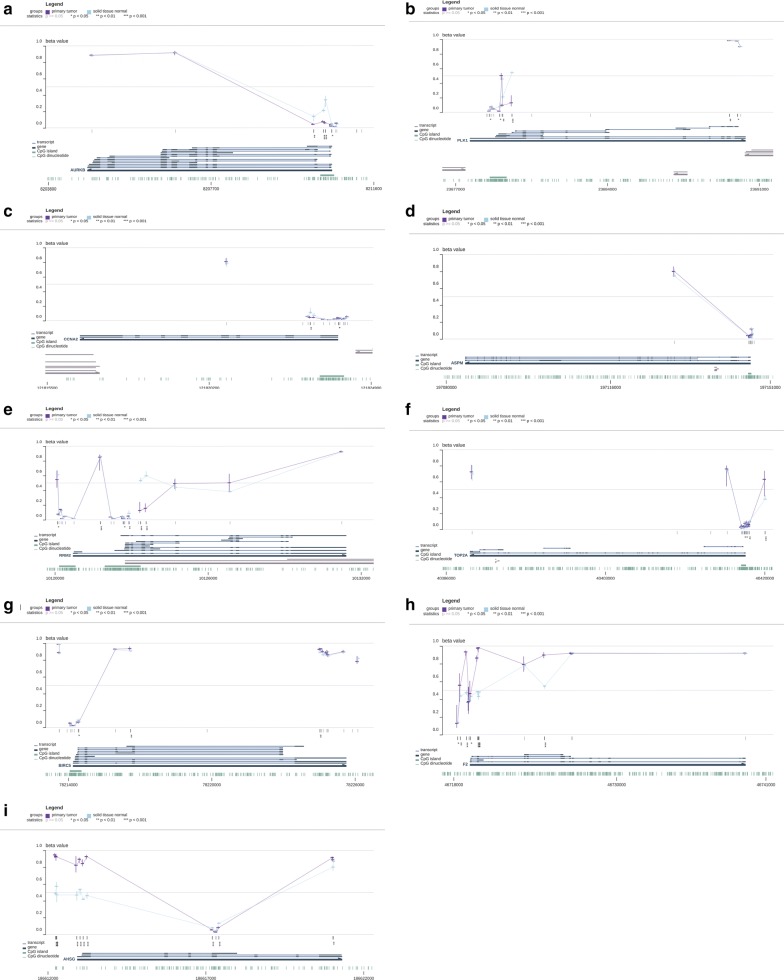
Verification of the methylation levels of 7 hypomethylated upregulated genes and 2 hypermethylated downregulated genes based on TCGA databases. **a** AURKB. **b** PLK1. **c** CCNA2. **d** ASPM. **e** RRM2. **f** TOP2A. **g** BIRC5. **h** F2. **i** AHSG

**Fig. 6:**
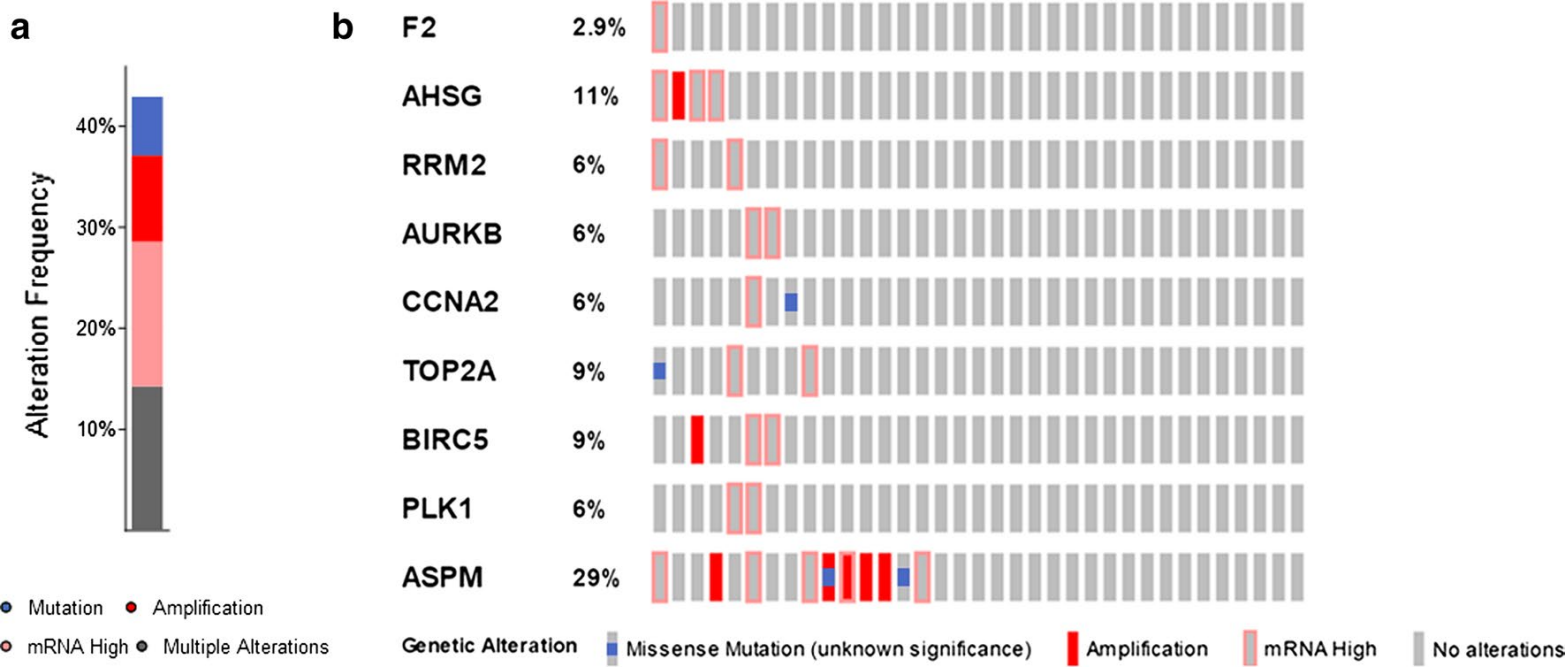
Genetic alterations of hub genes in the TCGA database. **a** Genetic alteration frequency of nine hub genes in 35 samples. Different colors represent different kinds of genetic alterations. **b** Summary of alterations per sample. Each sample is presented in a column with each gene in a row. Different kinds of genetic alterations are highlighted in different colors

To further investigate the identified hub genes, the TCGA database was used to validate our results. The expression and DNA methylation data of nine hub genes were obtained using MEXPRESS. The expression levels of seven hypomethylated upregulated genes and two hypermethylated downregulated genes were significantly different in the TCGA database, which were consistent with our results (Table 3). Except for ASPM, the methylation levels of these hub genes were consistent with our findings (Fig. 5a–i: AURKB, PLK1, CCNA2, ASPM, RRM2, TOP2A, BIRC5, F2, and AHSG). In addition, the presence of genetic alterations in the hub genes was examined using the cBioPortal tool. More than 40% of the patient tumors had at least one hub gene alteration and ASPM (29%) was the most frequently altered gene of the nine queried genes (Fig. 6).

**Table 3:** Verification of the expression levels of 7 hypomethylated upregulated genes and 2 hypermethylated downregulated genes based on TCGA databases

Category	Gene ID	logFC	P value
Upregulated	TOP2A	4.343206	2.81E−27
Upregulated	PLK1	4.137076	2.59E−24
Upregulated	BIRC5	3.999543	5.92E−23
Upregulated	RRM2	3.512025	2.17E−20
Upregulated	AURKB	4.000693	2.92E−20
Upregulated	CCNA2	3.299934	3.11E−19
Upregulated	ASPM	2.892425	1.85E−12
Downregulated	AHSG	− 6.23907	1.1E−09
Downregulated	F2	− 5.61955	1.31E−09

## Discussion

Cholangiocarcinoma (CCA) is a fatal malignancy that arises from cholangiocytes. In recent years, the incidence of CCA has risen, and CCA has become a major health burden worldwide [25]. The majority of patients with CCA present with unresectable or metastatic disease at the time of diagnosis because of the lack of specific symptoms or sensitive indicators [26]. Conventional radiotherapy and current chemotherapy treatments are not very effective [2]. Given the diagnostic difficulty and limited treatment options, the prognosis for patients with CCA is extremely poor [27]. Therefore, identifying novel molecular biomarkers and understanding the underlying mechanisms of carcinogenesis and progression in CCA are critically needed.

DNA methylation is a central epigenetic modification that plays a key role in cellular processes and generally occurs on cytosines that precede a guanine nucleotide [28, 29]. Typically, these cytosine–phosphate–guanine (CpG) sites are concentrated in large clusters (i.e., CpG islands) that are enriched mostly in the promoter region of genes [30]. Because the binding sites for many transcription factors are GC-rich, CpG islands are likely to enhance binding to transcriptional start sites [31]. CpG islands may also enhance the accessibility of DNA by regulating the chromatin structure. Methylation of CpG islands can impair transcription factor binding, recruit repressive methyl-binding proteins, and stably silence gene expression [31]. Thus, CpG islands are normally unmethylated in transcriptionally active genes and methylated in the promoters of silenced genes. To date, numerous studies have demonstrated a crucial role for both hypermethylation of tumor suppressor genes and hypomethylation of oncogenes in cancer development and progression [30]. For example, Wu et al. [32] found that JMJD2C could enhance the metastasis of colorectal cancer by decreasing the histone methylation of the MALAT1 promoter, thereby upregulating MALAT1 expression and enhancing the activity of the β-catenin signaling pathway. Furthermore, Liang et al. [33] identified many novel methylation-regulated differentially expressed genes involved in colon cancer tumorigenesis and progression by identifying hypermethylated downregulated genes and hypomethylated upregulated genes.

The initiation and progression of CCA is a complex and multistage process regulated by both genetic and epigenetic alterations [34]. An increasing number of studies have demonstrated the crucial biological functions of epigenetic modifications, especially DNA methylation, in CCA. For example, significant differences in the methylation levels of OPCML and HOXD9 were observed in serum cell-free DNA from CCA patients, and these two genes can be used for the differential diagnosis between cholangiocarcinoma and other biliary diseases [35]. Moreover, Wang et al. found that DANCR could bind EZH2 and modulate the histone methylation of the FBP1 promoter, thereby regulating the proliferation and migration of CCA cells [25]. However, previous studies mainly focused on specific genes with aberrant DNA methylation or individual gene methylation profiling microarrays. However, a systematic conjoint analysis involving both gene expression and methylation profiling in CCA may yield more accurate and reliable results. Thus, we performed an integrated bioinformatics analysis based on both gene expression and gene methylation profiling (GSE119336 and GSE38860, respectively) to screen for novel biomarkers and therapeutic targets in CCA for future research.

In the present study, we identified a total of 93 hypomethylated, upregulated genes and 98 hypermethylated, downregulated genes by integrating the DEGs and DMGs. Functional and pathway enrichment analysis indicated that the hypomethylated, upregulated genes were mainly enriched in the biological processes of the cell cycle, mitotic cell cycle checkpoint, mitotic sister chromatid separation, spindle assembly, nuclear division, and cell division. These findings are reasonable because it is well known that dysregulation of cell cycle progression, including nuclear division, sister chromatid separation, and spindle assembly, is closely related to cancer 1 [36–39]. Specifically, we identified many upregulated MeDEGs that participate in cell cycle progression, including BIRC5, PLK1, SGOL1, and ASPM. Some of these MeDEGs are involved in tumorigenesis. For instance, SGOL1, a member of the shugoshin family of proteins, is thought to protect centromeric cohesion during mitosis, and its dysregulation at centromeres can lead to chromosome missegregation and miotic arrest [40, 41]. Mu et al. found that SGOL1 expression levels are higher in prostate cancer tissues, and SGLO1 knockdown results in the inhibition of tumor cell proliferation, migration, and invasion [42]. In addition, SGOL1 has been found in other cancers (e.g., breast cancer and glioblastoma) [43–45]. However, few studies have described the methylation state of SGOL1, and research on SGOL1 in CCA has been rarely reported. Therefore, further research on the relationship between these MeDEGs and the cell cycle is warranted.

Pathway analysis also revealed that the hypomethylated, upregulated genes were enriched in MST1 signaling, which has demonstrated significant effects in multiple types of human cancer. MST1 can bind to its specific receptor MST1R, and MST1/MST1R signaling plays an important role in regulating inflammation and stimulating chemotaxis and phagocytosis [46]. The upregulation of MST1R promotes the progression of many epithelial cancers, including pancreatic, lung, and breast cancer [46–48]. We previously demonstrated that MST1R is upregulated and associated with overall survival in CCA [49]. However, the underlying mechanisms regulating MST1R remain largely unknown. In the current study, we found that MST1R was hypomethylated in CCA, which provides a foundation for future research.

The 98 hypermethylated, downregulated genes were mainly enriched in the biological processes of carboxylic acid metabolism, negative regulation of proteolysis, cytolysis, and xenobiotic and monocarboxylic acid metabolism. We also identified the involvement of complement and coagulation cascades, bile secretion, drug metabolism, cholesterol metabolism, and CYP2E1 reactions with these genetic changes. Both the biological processes and pathways analysis showed an abundant enrichment in metabolism, particularly bile metabolism. Among these genes, we found ABCB11, which encodes the bile salt export pump (BSEP), was involved in bile secretion, cholesterol metabolism, bile acid and bile salt metabolism, and recycling of bile acids and salts based on the pathway analysis. BSEP mediates the secretion of bile acids across the canicular membrane of hepatocytes into bile to provide the osmotic pressure for bile flow [50, 51]. Strautnieks et al. found that BSEP is typically absent or greatly reduced due to ABCB11 mutations, and 15% of patients with BSEP deficiency developed hepatocellular carcinoma or CCA [52]. BSEP deficiency may cause CCA through bile-composition shifts or bile-acid damage within cells capable of hepatocytic or cholangiocytic differentiation [53]. Moreover, previous studies also demonstrated a critical role for ABCB11 in lung and ovarian cancer [54, 55]. However, both Srimunta et al. and Fujikura et al. did not find differentially expressed BSEP levels in CCA tissues using immunohistochemistry or real-time RT-PCR, which is inconsistent with our findings [56, 57]. Therefore, further investigation is needed. The cytochrome P450 (CYP) enzymes are membrane-bound hemoproteins that play a pivotal role in the natural product biosynthesis, drug metabolism, cellular metabolism and homeostasis [58, 59]. Anti-cancer drugs and herbs undergo metabolism mediated by CYP enzymes in the body to form numerous stable metabolites and this metabolism may significantly alter their therapeutic potential [60]. Besides, several studies have demonstrated the important role CYP enzymes played in CCA progression. For example, Zhang et al. found that 1,2-dichloropropane, a carcinogenic paint remover, could influence the proliferation and apoptosis of cholangiocytes and this effect is mediated through CYP450 [61]. Moreover, Khenjanta et al. found that CYP39A1 was down regulated in 70% of CCA patients and low expression of CYP39A1 demonstrated a significant correlation with metastasis [62]. However, the mechanisms of methylation regulation mechanism for these CYP enzymes are still unknown.

In the PPI networks generated for hypomethylated, upregulated and hypermethylated, downregulated genes, significantly more interactions than expected were observed with a PPI enrichment *P* value < 1.0e−16, which indicates that these genes are biologically connected. Additional analysis identified nine hub genes (F2, AHSG, RRM2, AURKB, CCNA2, TOP2A, BIRC5, PLK1, and ASPM), which were validated using the TCGA database. Many of these genes are associated with CCA. For example, Shen et al. found that AURKB is overexpressed in CCA and correlates with overall survival and tumor grade [63]. AURKB is also upregulated and hypomethylated in hepatocellular carcinoma [64, 65]. However, aberrant methylation of AURKB in CCA has not been previously reported.

We explored potential genetic alterations of the identified hub genes using cBioPortal. We observed that more than 40% of the patient tumors analyzed had at least one hub gene alteration. Of the nine evaluated genes, ASPM (Abnormal Spindle Microtubule Assembly) was the most frequently altered (29%). The ASPM protein is involved in mitotic spindle regulation and coordination of mitotic processes [66]. Previous studies have shown that ASPM plays an essential role in tumorigenesis and the development of numerous types of cancers, including pancreatic and breast cancer and clear cell renal cell carcinoma [67, 68]. However, the mechanisms of methylation regulation for ASPM and the role of ASPM in CCA are not known.

Several limitations to the present study should be mentioned. First, our research only focused on upregulated, hypomethylated genes and downregulated, hypermethylated genes. However, contra-regulated genes were not included and need to be studied further. Second, our study was limited to the data of bioinformatics arrays and tools. We did not investigate clinical parameters and prognosis, which may reduce the reliability of our findings. Third, although we validated the identified hub genes using the TCGA database, biological experiments were not performed. Our future research will focus on experimental verification of these results. Finally, we obtained the MeDEGs by overlapping the DEGs and DMGs from two different datasets (expression dataset GSE119336 and DNA methylation dataset GSE38860) but did not include a dataset that included both expression and methylation data for CCA. In addition, the numbers of samples and datasets for the DEG and DMGs were small, which could introduce false positives and reduce the reliability of our findings. Therefore, an independent expression and methylation study for CCA that includes large-scale multicenter clinical samples should be carried out.

## Conclusions

In conclusion, using an integrated bioinformatics analysis, our study identified methylation-regulated differentially expressed genes and explored their related pathways and functions in CCA. In addition, we constructed a PPI network that identified nine hub genes. Our findings may deepen the understanding of the methylation-mediated regulatory mechanisms underlying CCA and provide some novel therapeutic targets for further research.

## Data Availability

The data used to support the findings of this study are included in the article.

## Abbreviations

CCA: cholangiocarcinoma
MeDEGs: methylation-regulated differentially expressed genes
GEO: Gene Expression Omnibus
DEGs: differentially expressed genes
DMGs: differentially methylated genes
PPI: protein–protein interaction
TCGA: The Cancer Genome Atlas
MCODE: Molecular Complex Detection
GO: Gene Ontology
KEGG: Kyoto Encyclopedia of Genes and Genomes
STRING: The Search Tool for the Retrieval of Interacting Genes
CpG: cytosine–phosphate–guanine
MGMT: O6-methylguanine-DNA methyltransferase
ASPM: Abnormal Spindle Microtubule Assembly
